# Ultrasonic image analysis of longitudinal strain in uremic patients with preserved left ventricular ejection fraction

**DOI:** 10.1186/s12938-018-0536-y

**Published:** 2018-08-20

**Authors:** Yuqin Ma, Bo Zhang, Yuzhen Zhang, Yun Dong, Ruiqing Zhang

**Affiliations:** 1 Department of Ultrasound in Medicine, Shanghai East Hospital, Tongji University, School of Medicine, Shanghai, 200120 China; 2 Key Laboratory of Arrhythmias of the Ministry of Education of China, Research Center for Translational Medicine, Shanghai East Hospital, Tongji University School of Medicine, Shanghai, 200120 China; 3 Department of Nephrology, Shanghai East Hospital, Tongji University, School of Medicine, Shanghai, 200120 China

**Keywords:** Speckle tracking echocardiography, Ventricular function, left, Strain, Uremia, Renal dialysis

## Abstract

**Background:**

Patients with uremia have high cardiovascular disease morbidity and mortality despite having normal left ventricular ejection fraction (LVEF). Longitudinal strain (LS) can be associated with subtle changes in LV systolic function. The aim of this study was to use two-dimensional speckle-tracking echocardiography (2DSTE) to assess subclinical LV myocardial dysfunction and to explore strain-changing regularities in uremic patients with LVEF ≥ 55%.

**Methods:**

The study population included 40 uremic patients and 40 healthy volunteers. 2DSTE was performed on all participants to assess peak LS in the basal, mid and apical LV (BLS, MLS and ALS) and the respective time to peak LS (T-BLS, T-MLS, T-ALS).

**Results:**

BLS, MLS, and ALS were significantly decreased in the uremic group relative to healthy controls and LS increased going in a basal to apical direction in both groups. T-BLS, T-MLS and T-ALS was significantly increased in the uremic group compared with the control group. In uremic patients, T-BLS, but not T-MLS or T-ALS, was significantly delayed relative to the control group. Bivariate analysis of creatinine (Cr) or urea nitrogen and strain parameters revealed a correlation only between ALS and Cr.

**Conclusion:**

2DSTE can identify LV myocardial abnormalities in uremic patients with preserved LVEF at early stage, as well as some changing regularities of LS and T-LS in the left ventricle.

## Background

Chronic kidney disease (CKD) is an important public health problem that is characterized by poor health outcomes and high health care costs [1]. Uremia is the end-stage of CKD and uremic patients have a higher risk of cardiovascular disease (CVD) than the general population as well as a higher mortality rate for cardiovascular events [2]. Moreover, uremic patients with CVD have a poor prognosis [3]. However, the underlying mechanisms for the higher incidence of CVD in uremic patients were not yet fully elucidated, they may be associated with uremic cardiomyopathy, a common complication that is characterized by cardiac fibrosis, capillary rarefaction, left ventricular hypertrophy, and both systolic and diastolic dysfunction [4]. In uremic patients, heart failure (HF) is the major cardiovascular complication and its incidence increases with declining kidney function [5]. CKD and CVD share common traditional risk factors, such as smoking, obesity, hypertension, diabetes mellitus, and dyslipidemia [6]. Current methods for assessment of myocardial dysfunction chiefly rely on visual wall motion analysis, which is subjective and limited by high observer variability in the estimation of left ventricular ejection fraction (LVEF) [7]. Indeed, approximately 50% patients with HF have a normal LVEF [8]. As such, CVD is often underdiagnosed and undertreated in uremic patients. Due to difficulties in diagnosis, clinicians may miss early treatment opportunities and the patient prognosis may be poor relative to cases in which the LVEF or visual wall motion is obviously descended. Therefore, novel indices to detect incipient myocardial alterations in uremic patients are urgently needed. Magnetic resonance imaging (MRI) has been the noninvasive and accurate technique for the detailed evaluation myocardial structure and function [9]. However, MRI had some disadvantages such as low time and spatial resolution, complicated post-processing, time-consuming and expensive inspection, which limits its clinical application [10]. Recently, two-dimensional speckle tracking echocardiography (2DSTE) has emerged as a promising method to assess LV systolic function. In contrast to M-mode or tissue Doppler velocity that cannot differentiate strain from passive movement, 2DSTE can measure strain as active wall thickening [11]. 2DSTE is based on frame-by-frame tracking of tiny echo-dense speckles within the myocardium and provides time-myocardial percent deformation curves across the entire LV that are divided into 16 segments throughout the cardiac cycle [12]. Among the 2DSTE parameters, LS is more reproducible and accurate [13]. Several studies demonstrated that LS is a sensitive indicator for detection of subtle disturbances in LV systolic function, and can quantitatively measure myocardial longitudinal contractility that is often impaired in patients with HF [14]. To date, most studies had focused on global longitudinal strain (GLS), and changes in the three left ventricle levels (basal, middle and apical) were examined less frequently. The objective of this study was to use 2DSTE to assess subclinical LV myocardial dysfunction and to explore strain-changing regularities of the LV in uremic patients with LVEF ≥ 55%.

## Patients and methods

### Study participants

Between March 2017 and December 2017, 40 clinically stable uremic outpatients undergoing regular hemodialysis (three times per week) at the Shanghai East Hospital nephrology clinic were consecutively enrolled. Patients having arrhythmia, primary hypertension, previous myocardial infarction, moderate or large hydropericardium, or LV systolic dysfunction with LVEF < 55% were excluded. The underlying causes of uremia among the study subjects were: chronic glomerulonephritis (16 cases), congenital renal malformation (4 cases), diabetes mellitus (7 cases), chronic interstitial nephritis (5 cases), polycystic kidney (4 cases), gouty nephropathy (2 cases) and purpura nephritis (2 cases). In addition, 40 age- and sex-matched healthy volunteers were enrolled as controls. All control subjects met the following inclusion criteria: no history of cardiac symptoms, hypertension or diabetes, normal physical examination, electrocardiogram, chest radiography, renal function and echocardiography results and no use of medication. Written and informed consent was obtained from all study participants. The study was approved by the Shanghai East Hospital ethics committee.

### Laboratory findings

The biochemical indices of uremic outpatients included blood creatinine (Cr) and urea nitrogen (Un) levels, which were evaluated at the hospital laboratory on the same day as the sample collection. Reference levels of Cr and Un were 58–110 µmol/L and 3.20–7.10 mmol/L, respectively, in accordance with standards used by the biochemistry laboratory of Shanghai East Hospital.

### Echocardiogram

All participants underwent an echocardiogram using a commercially available echo system (Artida, Toshiba Medical Systems, Tochigi, Japan) with a dedicated software package (Ultra Extend, Toshiba Medical Systems). Baseline transthoracic echocardiograms were performed with a frame rate of 40–60 Hz. Patients or controls with insufficient two-dimensional (2D) image quality for subsequent analyses were excluded. All image acquisition was performed with the ECG placed at the left lateral decubitus position. All echocardiographic studies were performed according to ASE-recommended guidelines [15] by an experienced echocardiographer (Yun Dong) who was blinded to the study groups.

## 1. Traditional echocardiography measurements

Standard two-dimensional echocardiography was performed to obtain conventional parameters in the parasternal long axis, namely, the left atrial diameter (LAD), LV septal wall thickness (LVST), LV posterior wall thickness (LVPT), LV diameter at the end of diastole (LVEDD), and the LV diameter at the end of systole (LVESD). Fractional shortening (FS) was then calculated using the formula: [(LVEDD − LVESD)/LVEDD] × 100. LV end-diastolic volume (LVEDV), LV end-systolic volume (LVESV) and LVEF were calculated from the apical four-chamber and two-chamber views using Simpson’s biplane method [16]. The early (E) and late (A) diastolic peak velocities of the mitral inflow and the early (e) and late (a) diastolic peak velocities of the posterior mitral annulus were measured by pulse wave Doppler and Doppler tissue imaging. Subsequently, the E/A ratio, e/a ratio, and E/e ratio were calculated to evaluate LV diastolic function.

## 2. Two-dimensional speckle tracking echocardiography

**Fig. 1:**
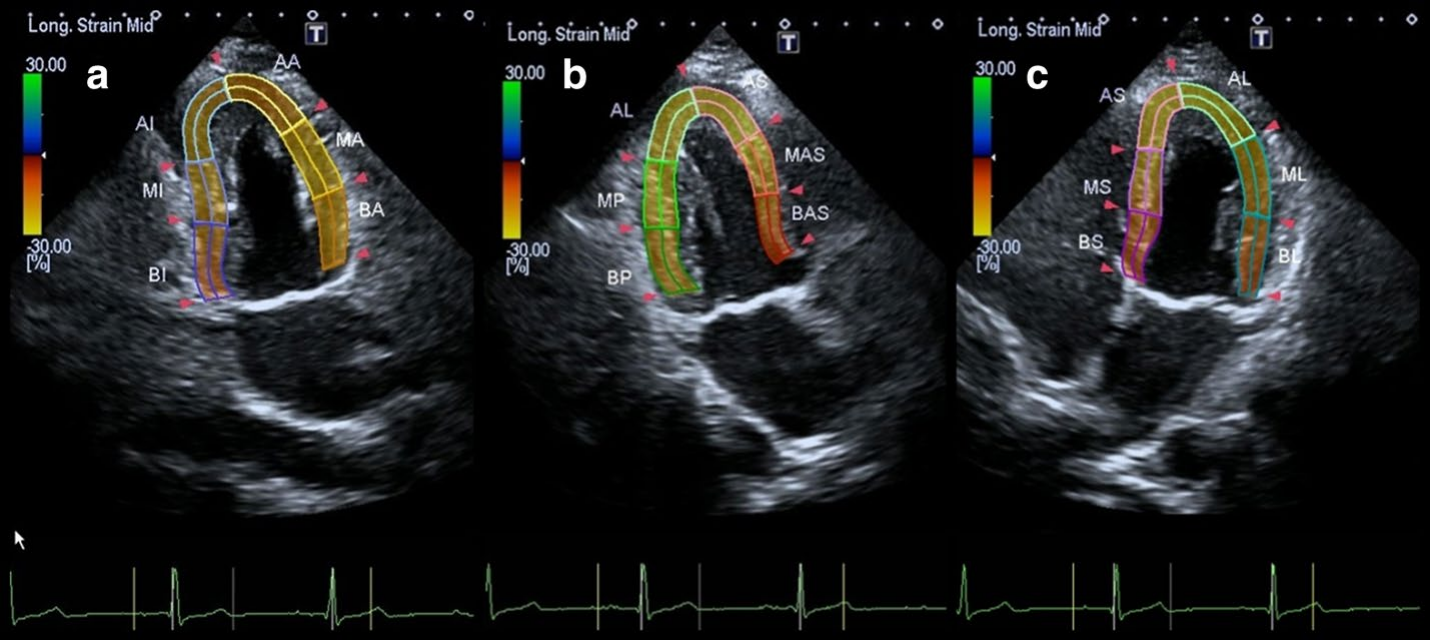
Example of 2DSTE long-axis image. **a**–**c** represent the apical 2-, 3- and 4-chamber view, respectively

**Fig. 2:**
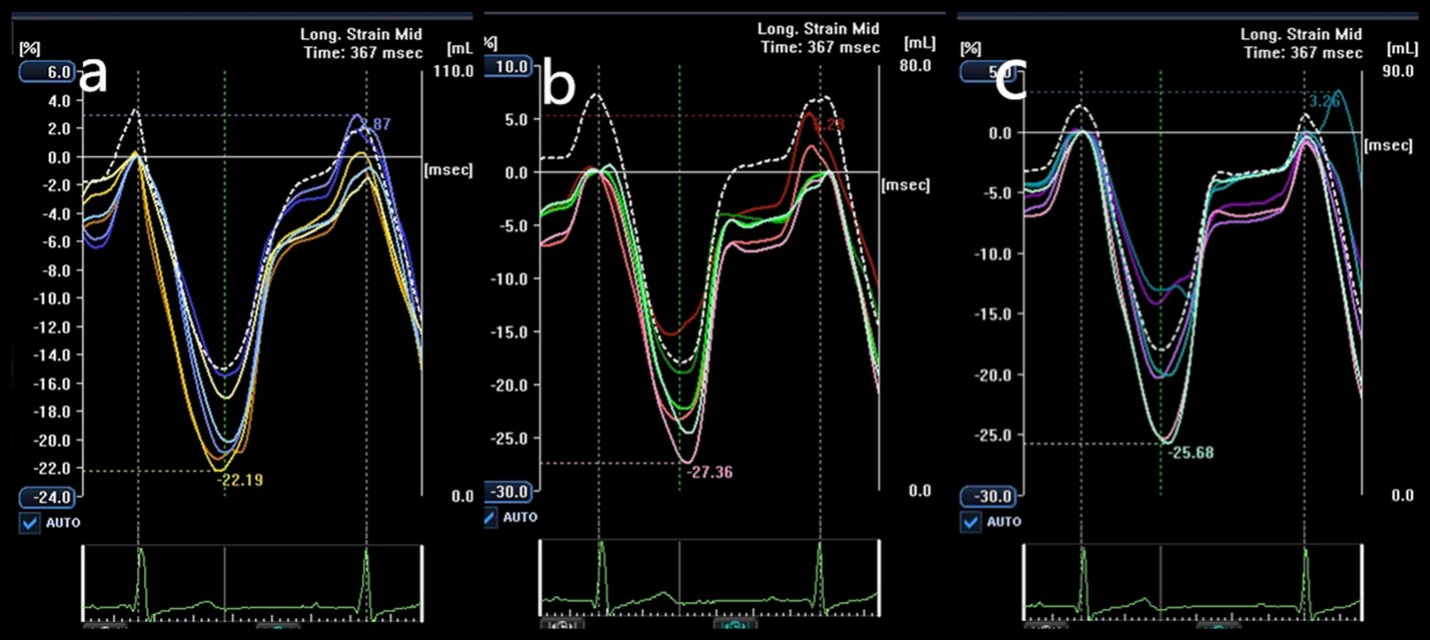
Example of 2DSTE time-longitudinal strain curves. **a**–**c** represent the apical 2-, 3- and 4-chamber view, respectively. The different color curves represent corresponding regional segments

Three standard apical views (i.e., 4-chamber, 2-chamber, and 3-chamber) were obtained over 4 cardiac cycles while the subject held his/her breath (Fig. 1). Temporal resolution was > 20 frames per cardiac cycle, depending on the heart rate. Frame rates were 50–60 Hz for gray-scale imaging used for speckle tracking. Sector width was optimized to allow complete myocardial visualization and maximize the frame rate. Gain settings were adjusted for routine gray-scale imaging to optimize endocardial definition. Two-dimensional speckle tracking analysis was performed online using customized 2D strain imaging software. After placement, the endocardial border was traced at the end-diastole (peak of QRS), and the epicardial border automatically appeared, which could be adjusted manually. The software then automatically tracked myocardial motion frame by frame and divided the LV wall into 16 segments wherein the LV was divided into 3 levels (basal, middle and apical) and the basal and middle LV were then subdivided into 6 segments each (anterior, inferior, lateral, posterior, septal, anteroseptal), whereas the apical region was divided into 4 segments (anterior, inferior, lateral, septal). Subsequently, time-longitudinal strain curves having 16 segments were automatically generated (Fig. 2). The 2DSTE parameters for this study were: basal, middle and apical peak LS (BLS, MLS and ALS) and their respective time to peak LS (T-BLS, T-MLS and T-ALS). Each parameter was calculated at the same level by averaging six segments from three apical views, namely 4-chamber view, 2-chamber view, and 3-chamber view.

### Intra- and inter-observer variability

Among the uremic and control groups, 5 subjects were randomly selected to test intra- and inter-observer variability in the 2DSTE analysis. The 2DSTE data for these subjects were reanalyzed 2 weeks after the first analysis by the same observer (Yun Dong) and by a second independent observer (Yuqin Ma). Both observers were blinded to the previous measurements.

## Statistical analysis

All analyses were performed using SPSS version 22 software (SPSS Inc., Chicago, IL, USA). Shapiro–Wilk test was used to assess the distribution of continuous variables. Results were expressed as mean ± standard deviation (SD) for normally distributed variables and median (interquartile range) for non-normally distributed variables. Comparisons between continuous variables were performed using Independent-Samples T test, Mann–Whitney U test, One Way Anova test or Kruskal–Wallis test as appropriate. Correlations between selected variables were verified by Pearson correlation coefficients (for normal data distribution) and Spearman coefficients (for non-normal data distribution). Intra-class correlation (ICC) of intra- and inter-observer variability in strain parameters were calculated. A P-value < 0.05 was considered significant for all described analyses.

## Results

Among the study subjects, 9 ERSD patients and 7 controls were excluded due to poor images that did not allow visualization of least three LV segments by 2DE. A total of 31 patients and 33 controls were included in the final statistical analysis. The possibility of acquiring high-quality images was 77.5 and 82.5% for the uremic and control group, respectively.

## 1. Conventional echocardiographic parameters

Conventional echocardiography parameters for the two groups are compared in Table 1. The uremic group had larger LAD, LVEDD, LVESD, LVEDV and LVESV, thicker LVST and LVPT as well as a higher ratio of E/e and e/a compared to the control group. Furthermore, FS and LVEF was decreased in the uremic group (P < 0.05).

**Table 1:** Conventional echocardiography parameters in uremic and control groups

Variable	Control	Uremic	P^*^
	n = 33	n = 31	
LAD (mm)	33.70 ± 3.01	37.64 ± 5.51	< 0.05
LVEDD (mm)	45.51 ± 4.10	47.93 ± 5.14	< 0.05
LVESD (mm)	28.92 ± 2.82	32.23 ± 5.84	< 0.05
IVST (mm)	8.76 ± 1.25	10.63 ± 1.90	< 0.05
LVPT (mm)	8.58 ± 1.17	10.15 ± 1.28	< 0.05
E/A	1.56 ± 0.70	1.03 ± 0.45	< 0.05
e/a	1.30 ± 0.49	5.46 ± 5.30	< 0.05
E/e	5.15 ± 1.07	7.57 ± 2.67	< 0.05
FS (%)	0.36 ± 0.02	0.33 ± 0.06	< 0.05
LVEDV (mL)	101.44 ± 20.29	114.50 ± 26.20	< 0.05
LVESV (mL)	37.03 ± 10.30	46.45 ± 16.84	< 0.05
LVEF (%)	0.63 ± 0.05	0.60 ± 0.07	< 0.05

*LAD* left atrial diameter, *LVEDD* left ventricular end-diastolic diameter, *LVESD* left ventricular end-systolic diameter, *IVST* interventricular septum thickness, *LVPWT* left ventricular posterior wall thickness, *E* transmitral peak velocity of early-diastole, *A* transmitral peak velocity of late-diastole, *e* velocity of early diastole of the mitral annulus, *a* velocity of late diastole of the mitral annulus, *E/A* the ratio of velocity of early and late diastole, *e/a* ratio of velocity of early and late diastole of mitral annulus, *FS* left ventricular fractional shortening, *LVEDV* left ventricular end-diastolic volume, *LVESV* left ventricular end-systolic volume, *LVEF* left ventricular ejection fraction

* Independent-Samples T test

### 2. 2DSTE parameters

**Fig. 3:**
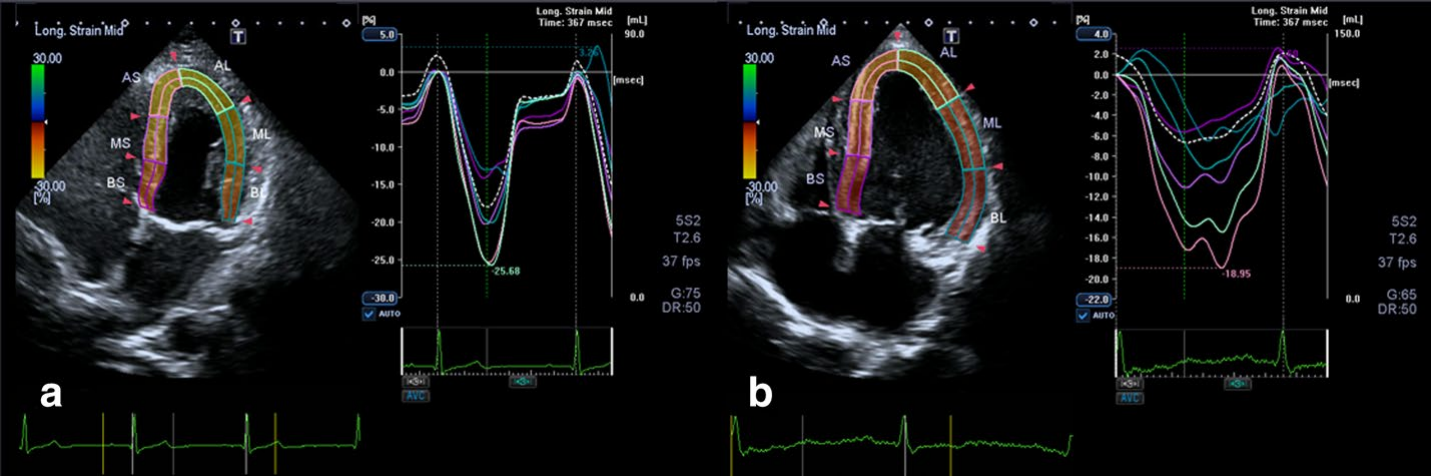
Representative comparison of time-longitudinal strain curves from apical 4-chamber views between control (**a**) and uremic (**b**) subjects. Relative to the image in **a**, the image in **b** image showed a decrease in peak LS and the time to the corresponding segmental peak LS was delayed
2.2. Intra-group analyses of BLS, MLS and ALS are shown in Table 3 and Fig. 4. From these results, we can conclude that both the patient and control group had a progressive increase in LS from BLS to ALS (P < 0.05).

2.1. The 2DSTE analyses for the two groups are shown in Table 2 and Fig. 3. Compared with the controls, uremic patients had significantly lower BLS, MLS and ALS. For time parameters, T-BLS, T-MLS, and T-ALS were significantly delayed in the uremic group (P < 0.05).

**Table 2:** 2DSTE parameters in the uremic and control groups

Variable	Control	Uremic	P*
	n = 33	n = 31	
BLS (%)	15.01 ± 1.65	12.84 ± 2.29	< 0.05
MLS (%)	17.44 ± 2.45	14.17 ± 3.19	< 0.05
ALS (%)	19.49 ± 3.60	17.08 ± 3.77	< 0.05
T-BLS (msec)	370.89 ± 41.08	433.52 ± 45.05	< 0.05
T-MLS (msec)	362.30 ± 29.80	415.05 ± 42.78	< 0.05
T-ALS (msec)	370.35 ± 28.09	398.53 ± 50.80	< 0.05

*BLS* basal peak longitudinal strain, *MLS* middle peak longitudinal strain, *ALS* apical peak longitudinal strain, *T-BLS* time to basal peak longitudinal strain, *T-MLS* time to middle peak longitudinal strain, *T-ALS* time to apical peak longitudinal strain

* Independent-Samples T test

**Table 3:** BLS, MLS and ALS in control and uremic groups

Variable	BLS	MLS	ALS	P^#^
Control	15.01 ± 1.65	17.44 ± 2.45	19.49 ± 3.60	< 0.05
Uremic	12.84 ± 2.29	14.17 ± 3.19	17.08 ± 3.77	< 0.05

*BLS* basal peak longitudinal strain, *MLS* middle peak longitudinal strain, *ALS* apical peak longitudinal strain

^#^One Way Anova test

**Fig. 4:**
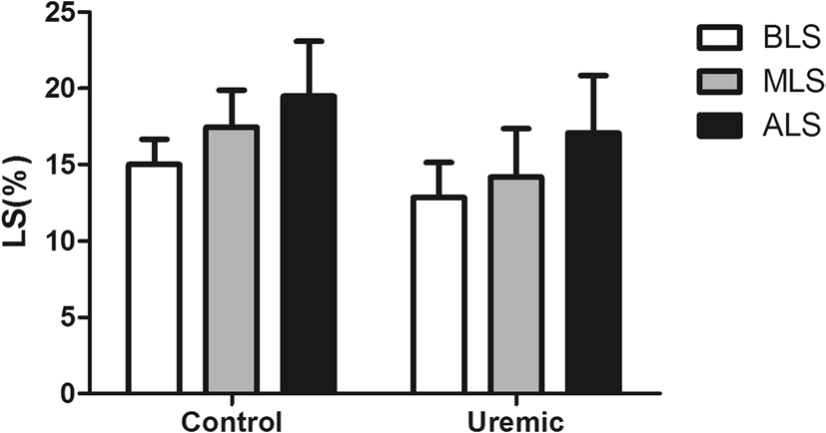
BLS, MLS and ALS in the control and uremic groups

**Fig. 5:**
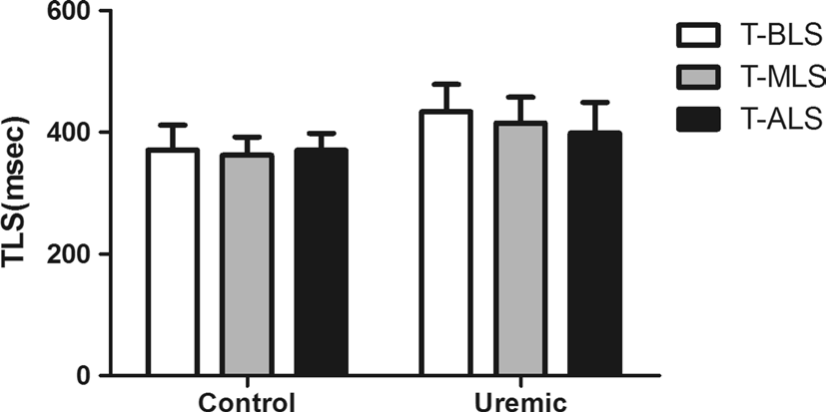
T-BLS, T-MLS and T-ALS in the control and uremic groups
  2.4. Relationships between 2DSTE parameters and biochemical indexes are shown in Table 5. The average Cr and Un levels in the uremic group were 824.71 ± 227.67 µmol/L and 22.75 ± 5.76 mmol/L. Bivariate analysis indicated that there was no favorable correlation between 2DSTE strain or time parameters and the biochemical indices (Cr and Un) except for the ALS and Cr, which showed a negative correlation (P < 0.05).

  2.3. Intra-group analyses of T-BLS, T-MLS and T-ALS are shown in Table 4 and Fig. 5. There were no significant differences among the control group (P > 0.05). Meanwhile, in the uremic group T-BLS, T-MLS and T-ALS notably differed and T-BLS was particularly prolonged compared to T-MLS and T-ALS (P < 0.05).

**Table 4:** T-BLS, T-MLS and T-ALS in control and uremic groups

Variable	T-BLS	T-MLS	T-ALS	P^#^
Control	370.89 ± 41.08	362.30 ± 29.80	370.35 ± 28.09	> 0.05
Uremic	433.52 ± 45.05	415.05 ± 42.78	398.53 ± 50.80	< 0.05

*T-BLS* time to basal peak longitudinal strain, *T-MLS* time to Middle peak longitudinal strain, *T-ALS* time to apical peak longitudinal strain

^#^One Way Anova test

**Table 5:** Correlation matrix between 2DSTE parameters, Cr and Un

Variable	BLS		MLS		ALS		T-BLS		T-MLS		T-ALS	
	R P^a^		R P^a^		R P^a^		R P^a^		R P^a^		R P^a^	
Cr	0.25	0.17	0.34	0.05	0.35	0.04	0.02	0.89	0.05	0.75	0.01	0.95
Un	0.06	0.71	0.02	0.91	0.16	0.37	0.12	0.50	0.01	0.94	0.10	0.58

*Cr* creatinine, *Un* urea nitrogen

^a^Pearson correlation coefficient
3. Intra- and inter-observer variability are shown in Table 6. The intraclass correlations (ICCs) were all above 0.8, indicating that the intra-observer and inter-observer reproducibility for the 2DSTE measurements were acceptable and satisfactory.

**Table 6:** Intra- and inter-observer variability

Variable	Intra-observer ICC	Inter-observer ICC
BLS	0.851	0.824
MLS	0.847	0.835
ALS	0.845	0.805
T-BLS	0.844	0.818
T-MLS	0.842	0.831
T-ALS	0.837	0.814

*BLS* basal peak longitudinal strain, *MLS* middle peak longitudinal strain, *ALS* apical peak longitudinal strain, *T-BLS* time to basal peak longitudinal strain, *T-MLS* time to middle peak longitudinal strain, *T-ALS* time to apical peak longitudinal strain

## Discussion

Many previous studies emphasized that the leading cause of death for uremic patients is CAD, including heart failure, myocardial infarction, and sudden cardiac death [17]. Thus, establishing reliable and non-invasive methods to detect cardiac structural and functional abnormalities are of great importance for effective treatment of uremic patients. However, LVEF and visual wall motion analysis by conventional echocardiography often do not reflect the LV systolic dysfunction at the early stage of disease [18, 19]. LVEF is not a simple and direct measure of LV systolic performance, which can be influenced by LV volume, preload, afterload, valvular function or regional hyper-kinesis after myocardial infarction [20]. 2DSTE can overcome some limitations of conventional echocardiographic measures to provide additional and quantitative information on myocardial strain indices in uremic patients [21]. Although tissue-Doppler imaging (TDI) also can measure LV strain and strain rate, such measurements can be limited by poor reproducibility, angle dependency and signal noise [22]. Several studies contended that 3DSTE is superior to 2DSTE for evaluation of LV dysfunction [23]. But the vital limitations of 3DSTE are lower temporal and spatial resolution, so the demands for patient’s imaging quality and experience of observers are higher than 2DSTE, which lead this technology can not apply to wide range of general patients during routine examination [24]. Based on all these reasons mentioned, in this study we evaluated the potential of 2DSTE for evaluating LV dysfunction in the uremic patient.

The major findings of our study were: (a) BLS, MLS, and ALS was significantly decreased in patient’s group compared with the control group, and LS increased from basal to apical in both groups. (b) T-BLS, T-MLS and T-ALS were significantly prolonged in the uremic group compared with the control group. In uremic patients, T-BLS showed a greater delay than did T-MLS and T-ALS, which were absent in the control group. (c) Bivariate analysis indicated that there was no correlation between strain parameters and Cr or Un except for ALS and Cr, which showed a negative correlation.

Longitudinal fibers are located mostly in the endo-epicardium, which is more sensitive to ischemia and pressure loading. LS mainly represents the function of subendocardial longitudinal myocardial fibers [25, 26]. Therefore, LS is often the first impairment in cardiac function that can be observed. In our study, the uremic patients had significantly lower LS and prolonged TLS compared to healthy volunteers, which was consistent with previous studies [27]. However, the mechanism responsible for abnormal LS and TLS in uremic patients has not been fully clarified. Impaired LS was reported to be associated with microvascular ischemia, interstitial fibrosis and cardiac myocyte hypertrophy caused by urotoxin, hypertension and hemodialysis-related myocardial stunning [28]. Cardiac workload is increased in uremia, which results from two separate pathways: pressure overload and volume overload. Pressure overload mainly derives from increased peripheral resistance and reduced arterial compliance due to sympathetic and renin-angiotensin system hyperactivity, hypertension, endothelial dysfunction, and vascular calcification/stiffening. Volume overload can be attributed to sodium and water retention, anemia, and the presence of an arteriovenous fistula in patients who have undergone hemodialysis for extended periods. Uremic toxins reportedly can inhibit the activity of succinate dehydrogenase in myocardial cells, which could both impede ATP production and promote mitochondrial swelling and fragmentation that in turn lead to metabolic imbalance and myocardial cell dysfunction [29]. Due to volume overload, elevated peripheral blood pressure and ATP deficiency, the time of LV contraction, namely TLS, lengthens to ensure sufficient ventricular ejection. Although the uniformity of LS from the base to apex of the LV varied across previous studies [30], in our study both the uremic and control groups had a progressive increase from BLS to ALS, which coincided with results from a study by Marwick et al. [31]. We assumed that such regularity arises from LV spatial structure differences: the basal LV is connected with the ascending aorta, whereas the apical region is independent. To the best of our knowledge, this study is one of the first to use 2DSTE to explore the changing regularity of TLS in uremic patients. The mechanism by which T-BLS alone is prolonged rather than that for the middle and apical regions in uremic patients awaits additional study as does the role of uremic toxins in contributing to decreased myocardial strain. In our study, bivariate analysis indicated no favorable correlation between 2DSTE parameters and the biochemical indices (Cr and Un) except for ALS and Cr. Most patients on maintenance dialysis therapy would be expected to have significantly decreased Cr and Un levels. These results could be associated with the small sample size or type of dialysis therapy used.

There were certain limitations to our study. First, our sample size was relatively small and conducted at a single center. Second, the patients were not followed over the long term to document the occurrence of cardiovascular events such as severe arrhythmias or ischemic events. Therefore, a larger study with long-term follow up of the study subject group is required to support our findings. Prospective clinical trials are also needed to establish whether our findings have therapeutic or prognostic implications and whether 2DSTE could be used to provide a better understanding of the physiopathology and treatment of uremic cardiomyopathy with normal LVEF.

## Conclusions

The findings in our study clearly demonstrate that 2DSTE can identify left ventricular myocardial abnormalities in uremic patients with preserved LVEF during the early disease stage, but we also found some changing regularity of LS and TLS in the left ventricle. These parameters may provide an opportunity to identify uremic patients who are at risk for future cardiovascular events. Large prospective, multicenter studies are needed to investigate these possibilities.
